# Redefining ‘normal’: A Canadian case study of cancer survivors’ experiences remaining and/or returning to work during the COVID-19 pandemic

**DOI:** 10.1371/journal.pone.0342935

**Published:** 2026-02-13

**Authors:** Carolyn Tran, Debbie Kane, Dale Rajacich, Kathryn Lafreniere, Caroline Hamm

**Affiliations:** 1 Department of Psychology, Faculty of Arts, Humanities, and Social Sciences, University of Windsor, Windsor, Canada; 2 Faculty of Nursing, University of Windsor, Windsor, Canada; 3 Department of Oncology, Windsor Regional Hospital, Windsor, Canada; Touro University California College of Pharmacy, UNITED STATES OF AMERICA

## Abstract

As cancer survival rates increase in Canada, a growing number of working-age individuals face challenges returning to work after treatment. This study examines the experiences of Canadian cancer survivors who remained in or returned to the workforce during the COVID-19 pandemic. Participants were cancer survivors aged 25−62 who had been employed prior to their diagnosis. They completed a brief online survey about their return-to-work (RTW) experiences and were invited to participate in a semi-structured interview. Seven participants took part in the interviews, which were transcribed and analyzed through inductive thematic analysis. Four overarching themes emerged: (1*)* The Perfect Storm of Systemic Challenges, highlighting healthcare barriers exacerbated by the pandemic; (2) You Are Not Alone, emphasizing the importance of social support in mitigating isolation; (3) One Size Does Not Fit All – Individual Journeys*,* reflecting the need for flexible workplace accommodations and patient self-advocacy; and (4) Creating a “New Normal,” illustrating how survivors reassessed priorities, work identities, and personal well-being. Participants described delayed treatments, lack of fertility options, and limited support during appointments due to pandemic restrictions. Workplace accommodations varied, with some survivors feeling supported while others faced inflexibility. Findings emphasized the need for improved healthcare responsiveness, personalized RTW accommodations, and improved employer training to support cancer survivors. The study also reveals how the COVID-19 pandemic exacerbated existing systemic gaps, underscoring the importance of preparing healthcare and employment systems to better support vulnerable populations during times of crisis.

## Introduction

It has been estimated that approximately two in five Canadians will develop cancer [1], and by 2031, it is projected that 2.2 million Canadians will be living with cancer [2,3]. Of those currently diagnosed with cancer, 42% are between the ages of 20 and 64 years, representing a substantial portion of the working-age population. While cancer survivors report a strong desire to return to work (RTW), they are 1.4 times more likely to be unemployed [4]. Further, on average, only 60% of cancer survivors return to work 1–2 years following treatment. As reported in the Ontario Cancer Care Plan 6 [2], as survival rates improve, there is a need to support cancer patients as they move from treatment to survivorship.

The ability to work is imperative for sustaining an individual’s identity [5,6]. For many, returning to work represents a return to normalcy, symbolizes recovery, raises self-esteem, and helps overcome the adverse effects of treatment [6–8]. The return to normalcy creates a positive impact on both physical and mental health for cancer survivors [9]. Thus, being employed or being able to RTW contributes to one’s quality of life [10].

A range of medical, occupational, and personal factors can influence survivors’ ability to RTW. Research has documented that reduced work ability was related to cancer type, type of treatment, health status, education, and physical limitations [11]. In a study analyzing the actions of managers that impact the RTW of breast cancer survivors, participants described breaches of confidentiality regarding their diagnosis and perceived discrimination related to the duration of sick leave [12]. Additionally, some survivors recounted being compelled to resign by their managers, being terminated, or not having their contracts renewed [12]. Generally, disease-related factors, such as the negative effects of treatment modalities, were found to be strongly associated with RTW [5]. Xu et al. emphasized the importance of addressing functional limitations, as many of these limitations are responsive to rehabilitation [9]. As a result, cancer survivors will be better equipped to meet the demands of their jobs. Moreover, a more widespread cancer can lead to increased functional deficits due to adverse effects of symptoms and complications from combined treatment modalities. This can also contribute to a greater burden of side effects [5] and, eventually, a less favorable prognosis. Consequently, this scenario may likely lead to challenges for RTW and employment for these individuals [13].

Workplace support has consistently been identified as a key facilitator in the RTW process [14]. Support from supervisors ranged from reducing responsibilities and developing graduated RTW plans tailored to the individual’s specific needs to expressing confidence in the employee’s capabilities [12]. The broader work environment, including coworkers and subordinates, also contributed to a supportive climate by fostering inclusivity, sharing workloads when necessary, and maintaining open lines of communication [6]. Occupational physicians often served as mediators between employees and employers, ensuring that medical recommendations were effectively translated into the workplace.

Treatment-related impairments also influence the experiences of individuals returning to work [9]. Several studies have found that fatigue and exhaustion impacted both home and professional work [11]. Beyond the physical health challenges, cancer survivors have expressed issues related to decreased income, lack of accommodations within the workforce, an inability to perform at their pre-diagnosis level, and not knowing where to seek information and support for further assistance [13]. These challenges may have been further intensified by the COVID-19 pandemic, which introduced additional stressors related to healthcare access, employment security, and workplace safety.

Existing research on RTW after cancer has focused on evaluating interventions and outcomes, often through quantitative designs and randomized controlled trials (RCTs). However, findings from this literature remain mixed. For example, RCTs have found no difference between intervention and control groups in time to RTW [15], whereas other RCTs suggest benefits of implementing an RTW intervention [16]. Additionally, a recent systematic review evaluating the effectiveness of non-medical interventions compared with usual care and no intervention concluded that RTW should be tailored to the patient’s needs [17]. While RCTs have advanced our understanding of what may be effective for RTW, there is limited insight into how cancer survivors experience RTW in an evolving social context, such as the COVID-19 pandemic.

Therefore, there is a need to improve understanding of survivors’ experiences of remaining in and/or returning to work. Using qualitative methods, this study had three main objectives: (1) to explore cancer patients’ experiences with remaining in the workforce while undergoing cancer treatment and/or returning to work upon completion of their treatment; (2) to identify supports and barriers that influence cancer survivors’ experiences remaining in or returning to work; and (3) to explore the impact of the COVID-19 pandemic on cancer survivors’ RTW experiences.

## Methodology

### Reflexivity and researchers’ positionality

The authors bring both direct and indirect experiences with cancer survivorship and RTW through family, friends, and professional roles. These experiences informed the study’s development and shaped decisions regarding the research design, participant recruitment, and data interpretation. The research team represented diverse disciplinary backgrounds, including health and community psychology (C.T. and K.L.), nursing (D.K. and D.R.), and medicine with expertise in cancer research and treatment (C.H.). While these backgrounds provided valuable contextual understanding, they also introduced potential interpretative biases. To enhance reflexivity and methodological rigour, we engaged in ongoing discussions throughout the research process by meeting regularly, using collaborative coding (D.R. and C.T.) and peer debriefing (D.K., D.R., K.L., and C.T.) to reflect on assumptions, challenge interpretations, and ensure that the analysis remained grounded in participants’ accounts.

### Study context

The original study idea was developed in 2019 to explore the experiences of cancer survivors returning to work following treatment. However, the world was faced with the COVID-19 pandemic, which resulted in unprecedented and widespread disruptions to workplaces, healthcare systems, and social norms. The pandemic prompted us to adapt the study’s focus to better reflect the realities that cancer survivors faced when returning to work during this time. As a result, this study included questions and stories about the impact of the COVID-19 pandemic on cancer survivors’ RTW experience. These changes to the study ensured that the research remained relevant to the evolving context in which the participants navigated during their RTW.

### Data collection

This exploratory study consisted of two components: an online 20-minute questionnaire about participants’ experiences with returning to work after their cancer diagnosis during the COVID-19 pandemic, followed by an opportunity to participate in an individual interview. Data collection for the brief survey began on December 1, 2021. Recruitment cards were distributed at a regional cancer centre, inviting individuals to participate in this study. To be eligible for this study, participants had to be between the ages of 25 and 62 and had to have been employed prior to an adjuvant cancer diagnosis. Individuals with a terminal cancer diagnosis were excluded, as they might not have anticipated returning to the workplace.

Within the online descriptive survey, we asked participants about their current employment status, the industry in which they worked, and whether the COVID-19 pandemic impacted their work situation. We also included open-ended questions to expand on how the COVID-19 crisis affected their ability to RTW and to allow them to share any additional comments they may wish to make. We ended the survey with a background questionnaire that asked about their demographic factors and disease characteristics, including cancer type, duration, and types of treatments.

Upon completing the online survey, a link was provided for those who wished to volunteer to participate in a telephone interview or via video chat. Participants were asked to provide their email address and phone number if they wished to be contacted for the interview portion of the study. Seventeen cancer survivors completed the questionnaire, and seven of those participants participated in the individual interview.

An interview guide was developed, including demographic information, diagnosis, treatment, employment history and questions such as: “Can you tell me how you made the decision to return to work?”; “If you needed accommodations when you returned to work, can you share with us what they were?”; “If there were particular challenges you faced as you worked during your cancer treatment (or upon return after treatment) can you share them with me?”; “How do you feel that continuing to work, or returning to work, has impacted your quality of life?” Additional prompts in the interview were informed by the responses that participants provided in the short descriptive survey, allowing the interviewer (D.R.) to probe deeper into the topics shared in the survey, such as why things felt difficult during the COVID-19 pandemic. D.R. conducted the interviews from August 23, 2022, to October 4, 2022. The interviews lasted 30–60 minutes.

### Participants

From the survey responses, seven participants, six women and one man, requested to be interviewed about their experiences with RTW after cancer treatment. The participants’ ages ranged from 29 to 59. All participants were married, and most participants were currently employed full-time. The majority of participants had been diagnosed with breast cancer (71.4%), while others reported diagnoses of endometrial cancer and chronic lymphocytic leukemia. Participants had undergone previous cancer treatments, such as surgery, chemotherapy, radiation therapy, and hormone therapy, within the past year or more. A more detailed list of participants’ background characteristics is provided in Table 1.

**Table 1:** Background Characteristics of Interview Sample.

Characteristics	*n*	%	*M*	*SD*	Range
**Age**	7	--	48.43	10.36	29–59
**Gender**					
Female	6	85.7			
Male	1	14.3			
**Ethnicity**					
White/European	6	85.7			
South Asian	1	14.3			
**Employment Status**					
Currently employed full-time	4	57.1			
Currently employed part-time	1	14.3			
Currently self-employed	1	14.3			
On long-term disability leave	1	14.3			
**Relationship Status**					
Married	7	100			
Living Arrangements*					
Live alone	1	14.3			
Live with spouse	5	71.4			
Live with children 18 and over	2	28.6			
**Level of Education**					
Community college diploma	2	28.6			
Bachelor’s degree	3	42.9			
Graduate or professional degree	2	28.6			
**Cancer type**					
Breast	5	71.4			
Endometrial	1	14.3			
Chronic Lymphocytic Leukemia	1	14.3			
**Months since diagnosis**	7	--	42.00	24.87	13–90
Previous treatments for this cancer*					
Surgery	6	85.7			
Chemotherapy	6	85.7			
Radiation therapy	5	71.4			
Hormone treatment	4	57.1			
**Current treatments for this cancer**					
Hormone treatment	4	57.1			
No current treatment	3	42.9			

Note. *May check multiple responses for this category.

### Ethical considerations

The University of Windsor’s Research Ethics Board approved the study (REB number: 39424). The participants were outpatients recruited from the local cancer centre. To obtain consent for the online descriptive survey, participants visited a Qualtrics link. They were presented with the consent form, which they signed to consent to the collection of their descriptive data. Participants were redirected to a separate page and were invited to provide their contact information for a follow-up interview at a scheduled date.

Participants who were interested in being interviewed were contacted by the researcher, D.R., who then scheduled an interview date. At the beginning of each interview, the researcher, D.R., read the consent form to all participants. D.R. ensured that informed consent was obtained before audio recording. During this process, participants were informed that they could withdraw from the study at any time during the interview. Participants provided oral informed consent. To document participants’ consent, D.R. started the recordings by thanking the participant for agreeing to participate. All interviews were audio-recorded for transcription and destroyed once the transcripts were completed. Participants were assigned pseudonyms to protect their identities.

## Data analysis & results

### Thematic analysis

An inductive thematic analysis was conducted to identify common themes in cancer survivors’ experiences returning to work during the COVID-19 pandemic. Researchers C.T. and D.R. collaboratively developed the initial codes from each participant’s interview. Meetings were held to review the initial codes and identify potential themes from participant responses. These codes were then shared with D.K. and K.L. Four overarching themes were identified, reviewed, defined, and named [18]. Each theme was found to have multiple subthemes, representing the complexity of each participant’s experiences during this time. Table 2 summarizes each theme and examples of representative quotes from participants’ responses.

**Table 2:** Thematic Table with Sample Quotes.

Theme	Sub-themes	Examples
The perfect storm of systemic challenges	Choosing one’s battle	I kind of got robbed of the decision of whether or not I wanted to have them [children]. *-Jennifer*
	Dealing with COVID-19 protocols	I would have two cell phones with me. If I could only go into appointments- appointments on my own. Um, my husband would be on one and my mother on the other […]. That was also a challenge, not being able to have people in the building with you. Even surgery was very difficult because you’re dropped off at the door and you go in on your own. And, um, and I’m sure there- there’s many people who experienced that. I’m not the only one, um, but everything was done on- on my own. Um, and- and then you go up to the parking lot and meet up with your loved ones. But yeah, that- *that* was challenging. Necessary. Necessary. I don’t- I don’t *disagree* with the decision from the hospital’s perspective, but definitely challenging from a patient perspective. -*Mary*
You are not alone	Support from social circle	But when you look back now. I think the intensity of it is […] time heals, and I think […] believing in the faith and having that, like, you know everyone like felt, you’re not alone, like a lot of people going through it. So, it’s something that you realized that you don’t say, ‘Oh, why me? No, why not?’ We all have tests or trials we go through. So, and uh- so, yeah, so it was kind of, you know, you don’t expect things like that, but then you say, ‘You know, we are all in this.’ Like, it can affect any one of us. So, yeah. *- Sara*
	Learning from each other	I have this vision where if all these young women or older women or any or all women or even men, if that’d be the case, uh, were able to meet and emphasize the importance of wellness and- and the big picture. So, what I mean by that is getting out and going for a walk, um, connecting with people. Don’t be afraid to share your story because there are many stories that can help you, and you can help them. *-Lynn*
One size does not fit all – Individual journeys	Patient self-advocacy	I took three days off work, um, and I just called every hospital, every plastic surgeon, every oncology department in Ontario. I even contacted private clinics and started just inquiring and, um, was met with, ‘You shouldn’t be calling here. This should be coming through your family doctor.’ And it was really attempting to just get an earlier, um, meeting with- with a team and to get further information and to, um, be able to be informed about what my choices are to make a decision to get surgery dates. *-Mary*
	The need for flexible workplace accommodations	The first time around, I didn’t know that I was going to need accommodations either. I felt, uh, I felt pretty together when I went in there. And it was only the repetition of the path that made me slowly aware that, ‘Wow, actually. Apparently, I have- Now I have joint issues that I wasn’t aware of.’ *-Joanne*
Creating a “new normal”	Self-reflection	I think… what I went through in regards to, like, my illness and just all the treatments and time off and time to just really re-evaluate my life. [...] I realized that I was dealing with a lot of stress before I was diagnosed with cancer. And it started coming back within the last year or so, and I started to ask myself why that was happening and what was causing it? And it was my place of work, unfortunately. Um, so I recently resigned to my position at that office, and I started a new position. *-Jennifer*
	New work conditions (for better or worse)	They are supportive, but I can see and sense the disappointment because I’m- I’m- I’ve been there 25 years, and I basically have the workload that I had when I was full-time. And I’m only doing it 3 afternoons a week. And I can see that they want, like, they want me to be there more. But I told them, I said, “You’re not going to get more out of me than you’re getting now.” Because I agreed to come back 20 hours a week, and it was too much. So now, then I went to 15 and with the lymphedema, I’ve been calling in, so I’m struggling to get 9 hours. So, and I really- I really feel like I should be off completely. But I- I like what I do, and I don’t want to disappoint anybody. *-Tracey*

### The perfect storm of systemic challenges

The COVID-19 pandemic gave rise to what was described as “the perfect storm” of systemic challenges within the Canadian healthcare system. One participant, Mary, explained how the pandemic highlighted the healthcare system’s already-existing weaknesses: “If they [the weaknesses] weren’t addressed in good periods, it obviously exacerbates during a period such as a pandemic.” Mary described the system as failing the patients, the doctors, and the teams that work in it. She acknowledged that the medical system is even weaker after the pandemic, and the healthcare team is emotionally and mentally exhausted. This theme had two subthemes highlighting the perfect storm of systemic challenges: choosing one’s battles and COVID-19 protocols.

#### Choosing one’s battle.

In this study, participants detailed their challenges with the local healthcare system during the COVID-19 pandemic. Systemic barriers included inflexible appointment times and limited accessibility to local healthcare. This situation compelled cancer patients to make critical decisions about their treatment options within a constrained timeframe.

Mary faced challenges with the healthcare system even before the pandemic, undergoing testing for eight years while local health experts misdiagnosed her breast cancer. Seeking help elsewhere, she eventually discovered her diagnosis at a different facility. During the COVID-19 pandemic, surgical delays added to her difficulties. She was given the option to have a mastectomy the following week. “It was something very quick, like, within a very short period of time,” she recalled. “And I remember saying that I have all these questions like, ‘Is that the right route to go?’” When asked if she could explore other options before the mastectomy, her surgeon told her significant delays would occur. Mary faced a crossroads: undergo a mastectomy immediately and remove the cancer or explore other options and risk the cancer growing for an undetermined period. She chose the latter.

In addition to inflexible appointments, the lack of local healthcare facilities significantly influenced one’s decision-making. Jennifer’s oncologist discussed reproductive alternatives with her, such as harvesting and fertilizing her eggs in vitro. However, the local fertility clinic was closed, and the nearest one was four hours away. When she contacted this clinic, they requested that she come in on the same day and stay for a week. Unfortunately, this schedule conflicted with her scans and tests for a tumour in her hometown, so she and her partner prioritized her health before worrying about any family plans.

In another case, Tracey, a nurse, explained how she knows how to navigate the [healthcare] system but also described it as difficult. As a nurse, Tracey is knowledgeable about what her body needs. “I know what I need to do. I know the supplies. I need to wrap my leg,” she continues, “And I can’t get some. I can’t get in to get a lymphatic massage and decompression until January. January!” She goes on to describe the supplies she needed and how she learned from YouTube how to do things on her own. Thus, she managed the swelling.

Due to the healthcare system’s lack of flexibility with appointments, patients were unfairly forced to make life-altering decisions without enough time to consider their options deeply. The urgency to battle cancer “robbed” patients of the opportunity to plan for the future. As a result, patients had to pick their battles and choose the best option for them, despite having to make sacrifices such as prolonging cancer growth or deciding not to have children.

#### Dealing with COVID-19 protocols.

COVID-19 protocols were implemented to reduce the transmission of COVID-19 between individuals. Such protocols included the shutdown of non-essential businesses, leading to furloughs or layoffs. Everyone was expected to practice social distancing, wear a mask, and thoroughly wash their hands. In addition to these changes, how health care appointments were handled also changed, including limiting them to the patient only.

Participants who were essential workers were faced with new protocols to follow. Sara, who was “kind of” prepared to RTW, was concerned about contracting COVID, especially given her compromised immune system. On the other hand, Tracey, who does research at the hospital where she worked, was asked to work in the COVID-19 emergency room. Her workplace deemed her research non-essential and needed their nurses to work the COVID frontlines. As a response, she was resistant to transferring to a different department due to her low blood count and requested to work from home.

Before the pandemic, patients could bring someone else to their appointments. For example, Glen, diagnosed before the pandemic, brought his wife or sister. He always had someone with him. Due to COVID-19 restrictions, patients had to attend their appointments alone to reduce the risk of transmission. Participants shared the difficulty of going to appointments alone. Mary found ways to work around the protocols by bringing two cell phones to her appointments, with her husband and mom on each line. Jennifer described feeling lonely during her treatments due to COVID-19 restrictions. Although they understood why the restrictions were in place, having close people there for support would have been nice.

Some positives stemmed from the COVID-19 protocols at the cancer clinic. Although Lynn’s husband or son used to go with her to her appointments, she admitted that sometimes it was best when they could not attend because it made the cancer clinic quieter. Additionally, the smell of food that other people brought in could be nauseating.

Participants shared how the COVID-19 lockdowns affected their mental health. Jennifer, diagnosed during the pandemic, was already laid off because her dental office was not working. Although dental offices opened in June 2020, she was going through chemotherapy and was not recommended to work during the pandemic. As a result, she had to stay in and was off work for nine months. “I started getting the itch to go back to work in November of 2020, and was supposed to start in January of 2021.” Because she was so antsy, her office welcomed her back earlier, and she worked on a modified schedule.

A contrast to everyone else’s stories was Glen. Glen just finished treatment right before the lockdown. He has been working from home for over 20 years, so he did not face any major challenges with COVID protocols:

I actually finished my last chemo treatment in February of 2020. It’s like, I got out of chemo. It’s like, ‘Hey Doctor, can I start seeing people again?’ Then COVID hit. So, a lot of people were complaining about not being able to see people. It’s like, well, I’ve already been through this for over six months.

### You are not alone

This theme represented the importance of not feeling alone during the COVID-19 pandemic. As mentioned in the earlier theme, COVID-19 protocols increased social distancing and isolation. Luckily, each participant had some form of support from the people in their lives to mitigate the effects of isolation and loneliness. Two subthemes were developed from this theme: support from social circle and learning from each other.

#### Support from social circle.

In this study, participants described the importance of having a strong support system to get through the hardships of cancer treatment. Most of the participants described their coworkers and colleagues as supportive. Lynn, a teacher, shared how she did not like to keep secrets from her colleagues and students, so she chose to disclose her diagnosis and was met with positivity:

I had a friend of mine that was kind of emotional when I first got the diagnosis. So, we had a staff meeting- we just happened to have a staff meeting a few days later. So, I had a friend of mine share with the staff the journey that I was expected to take. Um, and I also let my students know that I’d be away for a little while. And I did explain to them I’ll be battling a cancer. But I definitely was going to come through it and see them soon. So, I let everybody know. I just don’t think there’s any secrets to be had. And it’s amazing. Like I said, it’s amazing how many students are saying, ‘Oh my God, my grandma, my uncle, my sister.’ And they share their experiences. And then to see me back there and strong and healthy, happy and healthy. I think it’s a good reminder that even though we go through these tough things that you guys can get through it. So, I shared everything.

JoAnne believed her coworkers were kind and supportive. Jennifer also described her support system as amazing. Her coworkers supported her throughout the process. For example, when she was undergoing chemotherapy, she would stop by the dental office to chat with her coworkers and catch up because she missed them. In Tracey’s case, her coworkers and physicians reminded her to tell them what she needed to make herself comfortable at work. Unfortunately, Glen did not feel supported by his employees due to his management position, so he did not answer this interview question.

Sara described multiple dimensions in her life that gave her support during her cancer treatment. She said how her coworkers always reached out during her treatment and that teams needed to be there for one another. Additionally, Sara was the only participant who shared that her faith helped her through difficult times while undergoing cancer treatment. She described how she was very involved in the Muslim community. When asked about her deep faith, Sara responded:

I think going through that, I used to be part of a group, like, we would read the Holy Book. And I didn’t stop that. I just kept doing it. So, […] I had a very good close group of friends who were part of that group, and they would make those prayers for you. They knew I was going through it.

#### Learning from each other.

Although this subtheme was underdeveloped, it is worth noting that cancer patients and survivors expressed a need to build community among themselves. Because of the COVID-19 pandemic, the participants felt lonely and did not know whom to share their experiences with. Participants believed they would have benefited if they could have connected with others going through similar experiences. Lynn shared how she helped her colleague who went through breast cancer and how she wished that she could have someone do the same for her.

The desire to share information may help cancer patients and survivors feel safe in their treatment journey. Jennifer recalled her girlfriend’s experience with cancer and how she was by her side. She also acknowledged that her girlfriend was a driving factor in her journey, and she would not have a positive attitude if it were not for her.

But I do think that, um, because of going through the breast cancer with my girlfriend, it helped me mentally for, um, preparing me for what to expect. There wasn’t as much, um, darkness. I knew- I knew kind of what I was in for. That definitely helped my perspective.

### One size does not fit all – Individual journeys

This study revealed that each participant’s journey was unique and irreplicable. When examining their experiences with cancer and their RTW during the COVID-19 pandemic, every individual had a special story. These journeys were influenced by various factors, including the level of support from friends, family, and their workplace, the type of cancer they had, and their prior knowledge of the healthcare system. Two common subthemes were developed from the data: patient self-advocacy and the need for flexible workplace accommodations.

#### Patient self-advocacy.

Due to systemic challenges and barriers to receiving healthcare during the COVID-19 pandemic, participants in this study shared the different ways they had to self-advocate. Participants advocated for themselves for various reasons, such as fearing the unknown effects of COVID and their cancer treatment, finding additional options for their cancer treatment, and seeking information to alleviate the effects of cancer treatment.

Due to the healthcare system’s attempts to reduce the transmission of COVID, there were significant delays in surgery times. As such, the reduced surgery time has led to mastectomy being the only option for cancer treatment. When Mary was faced with the choice of getting a mastectomy or allowing the cancer to continue to grow for an undetermined amount of time, she chose the latter. She decided to self-advocate because she knew she had more options.

Mary could not get advice soon enough through the healthcare system, so she paid a plastic surgeon who had a private clinic from a different city to discuss her options for surgery. During her journey, she wrote letters to multiple people, including the hospital’s patient advocacy sector, her surgeon, and her family doctor. Mary faced many challenges, including not receiving responses or having unpleasant interactions. Despite being exhausted, she described herself as being persistent and acknowledged the sadness she felt for other patients going through cancer who would not have the energy to self-advocate.

Similarly, Tracey self-advocated for her health at work and was moved to a work-from-home setting rather than working at the hospital. She was concerned about not having an N95 mask when she had to go to work. Tracey recalled physicians giving out KN95 masks, and she needed N95 masks because she was face-to-face with patients and had low blood counts. Thanks to her research supervisor, she received N95 masks to ensure her safety. Other displays of self-advocacy included requesting reduced or modified work hours.

#### The need for flexible workplace accommodations.

The stories participants shared about their RTW experiences after surviving cancer exemplified the need for flexible workplace accommodations. Based on these interviews, some workplaces were very flexible in accommodating their employees’ needs, while others were not. Without flexibility in accommodations, participants found going back to work challenging. Some requested accommodations included modified and/or reduced work hours, ergonomic furniture, and a different work model, such as working from home.

Most participants, except Glen, had received some form of modified or reduced work hours. Sara attempted to work the hours her workplace assigned her, which were the new extended hours. Due to her fatigue, she requested to work only one weekend evening shift. She described her supervisor as being very kind and accommodating. As a dental hygienist, Jennifer also had a modified schedule, seeing one or two patients a week. On the other hand, Tracey’s work hours were reduced to 9 hours per week, and she was given a sit-to-stand desk.

Despite some accommodations being met, participants still faced barriers. Some participants did not realize they needed accommodations until afterward. When JoAnne returned to work at a manufacturing job, she realized her body could not perform the work. She tried to give her workplace options to help her, but they ignored her requests. Mary also had a similar experience to her RTW. She shared her frustration about how workplace accommodations needed to be modified. At first, she assumed she did not require accommodations until she developed pain, fatigue, and joint issues as a side effect of her medications.

Despite Mary’s reduced hours, she acknowledged that her workplace is deadline-driven, so the modifications do not feel like true modifications. She was not prepared to RTW. Although her workplace supported her, the demands of her job exceeded the support she was offered. Additional concerns about workplace accommodations included the need for workplaces to understand cancer survivors’ need for time off for appointments, as shared by Lynn and JoAnne.

### Creating a “new normal”

While undergoing cancer treatment during the COVID-19 pandemic, cancer survivors faced challenges and obstacles related to the changes they had to make when they returned to work. During the participant’s journey, they had to create a “new normal” for themselves. The “new normal” cancer survivors faced was shaped by their self-reflection on their cancer journey and the new work conditions that were, for better or worse, for them.

#### Self-reflection.

Participants in this study reflected and re-evaluated their lives and considered how they would have changed things. Some reflected on their workplace conditions and whether they could handle the workload. For example, Sara shared how she felt conflicted about returning to work and had moments of re-evaluation. She questioned her strength and whether she could handle the stress.

One participant, Jennifer, eventually quit her job and pursued something else. She had time off and explained how she had to re-evaluate her passion for her career. Her time off made her realize that she was dealing with a lot of stress before she was diagnosed with cancer. Upon reflection, she realized her workplace was the source of most of her stress. She sought a different job, and her mental health improved.

Reflecting on her experiences, Sara learned to appreciate things and not worry much about them. When she was first diagnosed with cancer, she faced uncertainty and doubt, which made her question the outcome of her journey. Moreover, she did not know much about chemotherapy at that time, so she wondered if there were different ways to treat cancer. Over time, Sara engaged in positive self-talk and celebrated the little wins to keep herself going: “You’re healthy. You’re OK. You’re doing what you can.” Glen also shared similar sentiments when he realized that stress could make things worse, “I’ve read some studies that said stress can lead to be a contributing factor in the cancer I had, and I vowed after that not to get stressed over anything, really.” As such, he did not work if he felt too tired.

Lynn and Mary wished they had known more about cancer. Lynn wished she knew more about lymphedema and had a friend who helped her with the needed information. Mary, who struggled for at least eight years to find a diagnosis, had to do her research. She read many research articles about dense breasts and breast cancer. In Mary’s self-reflection, she believed she did everything right; she regretted not insisting on an MRI earlier:

I did my personal breast exams. I discovered the lump. I reported it. I went for testing, and then I feel like I didn’t have enough knowledge to advocate, but I shouldn’t have to advocate for myself. To say I need an MRI because I do have dense breasts, and this is getting larger, and I don’t know why we keep doing the same test year, after year, after year, to say it’s benign and finding out later it was not benign and that it grew for years. And so that would be the one thing I would do different.

#### New work conditions (for better or worse).

Participants in this study shared their new work conditions, whether for better or worse. Some contemplated quitting, while others wished they could work at their full capacity. Most participants mentioned having to work reduced hours. Despite the reduced hours, participants shared their struggles.

Sara was glad she did not quit her job. She was given reduced work hours to accommodate her fatigue when she returned. Despite her long workdays, returning to work improved her quality of life. However, Sara acknowledged that working full-time hours would make returning to work more challenging. Sara described her work experience as giving her a purpose. Similarly, Jennifer shared the same sentiments; she loved being a dental hygienist and said, “I want to feel like a human again, where I’m an active part of this society.”

Mary described her RTW experience during the COVID-19 pandemic as “COVID PTSD.” She described knowing the hardships that her team members went through, such as family members passing away and not being able to see them. She described the disconnect that has happened to people due to the pandemic and the difficulties in showing empathy and compassion when one’s energy is depleted and in survival mode. Mary enjoyed her job and what she did, but at times, she questioned herself. She shared that she considered resigning weekly because her workload exceeded the accommodations. Similarly, Tracey worked as a nurse for 25 years and now struggles to work 9 hours per week. She explained that she could sense her coworkers’ disappointment.

On the other hand, Glen and Jennifer found benefits from the COVID-19 pandemic. Both participants realized there were other options to improve their happiness. For example, returning to work led Jennifer to recognize that her unhappiness stemmed from her job, so she sought work elsewhere and described herself as much happier. During the pandemic, Glen faced the struggles of his business and decided to adopt a different hiring practice, such as hiring outside his local region. Additionally, his business improved immensely thanks to the work-from-home model. He explained that he has employees across Canada, including in Edmonton, Calgary, and Montreal. Both these participants found a solution to the stressors of the pandemic.

## Discussion

Our study highlights the complex nature of cancer survivors’ return to work (RTW) during the COVID-19 pandemic. Cancer patients’ RTW experiences were shaped by their interactions across multiple ecological systems, including their immediate interpersonal contexts and broader societal structures. The findings of our study aligned with the ecological systems theory, which posits that an individual’s psychological state evolves through engagement with and immersion within distinct environmental contexts [19,20]. These findings demonstrated that RTW is not an individual effort, but a process shaped by interactions among personal, organizational, and societal systems.

At the individual level, the *microsystem*, participants described navigating physical limitations, cognitive challenges, and shifts in self-concept while seeking to restore a sense of normalcy and self-worth [6,7]. The next level, the *mesosystem,* refers to the connection between multiple microsystems. Supportive supervisors and employers who provided workplace accommodations such as flexible scheduling, reduced hours, and modified workstations, facilitated successful RTW and continued employment [6,14]. Conversely, those who did not receive adequate workplace accommodations felt the challenges of returning to work [6,12].

Beyond the workplace, participants’ experiences were also influenced by social structures, the *exosystem*. These included factors such as included healthcare accessibility, financial concerns, and community support systems that shaped their RTW experiences [11]. Moreover, the *macrosystem,* which involves the larger societal and cultural context (i.e., COVID-19 pandemic), introduced unique stressors that amplified existing inequities and barriers. As a result, participants reported delayed medical care, reduced autonomy in treatment decisions, and increased social isolation.

When viewed through this ecological lens, RTW emerges as a dynamic process shaped by interconnected systems that can either facilitate or hinder adaptation to RTW. The context of the pandemic further underscores the *chronosystemic* influence of time, indicating how external crises interacted with survivors’ personal recovery trajectories to create new vulnerabilities and opportunities for growth.

Understanding RTW in this way emphasizes the need for multilevel interventions that extend beyond individual coping strategies. Improving work reintegration should involve collaboration among employers, healthcare systems, and policy frameworks that collectively foster inclusive, flexible, and sustainable work environments for cancer survivors.

### Study limitations

While this study provides valuable insights into Canadian cancer survivors’ experiences of remaining and returning to work during the COVID-19 pandemic, several limitations should be acknowledged. The small sample size of seven participants, combined with the relative homogeneity in ethnicity, socioeconomic background, and cancer type (most being breast cancer survivors), may limit the transferability of the findings. In addition, participants were generally well enough to remain in or RTW, introducing a potential selection bias that excludes the perspectives of those facing greater health-related or socioeconomic barriers. As such, the results should be interpreted as exploratory. Future research could strengthen these findings by recruiting participants from larger cities with greater ethnic diversity. Moreover, direct recruitment across different workplace settings might improve our understanding of the experiences of individuals from different work sectors, cancer types, and health statuses, thereby providing a more comprehensive picture of RTW experiences.

Finally, because this study was conducted within a single region of Southwestern Ontario, the findings may reflect local healthcare and workplace conditions, as well as pandemic-related factors, which may differ from those in other settings. Expanding future research into multiple regions or national samples could help assess the transferability of these findings. Despite these limitations, the study offers important regionally grounded insights that can inform future research and workplace interventions to support cancer survivors’ work reintegration.

## Conclusion and practical implications

### Improving patient experiences with the healthcare system

This study highlights the need to address gaps in the healthcare system to prevent problems from worsening during crises such as the COVID-19 pandemic. It is difficult for cancer patients to undergo cancer treatment, in addition to navigating an already difficult healthcare system. It is essential to reassess the accessibility of our healthcare system for individuals battling cancer. For future crises, the healthcare system could prepare by establishing clear protocols to ensure the continuity of cancer care, enabling patients to maintain access to screening, treatment, and rehabilitation. Healthcare providers should also receive improved guidance and training to support survivors’ RTW and coordinate closely with employers to facilitate it.

### Improving cancer survivors’ RTW experiences

This study highlights the importance of flexibility in workplace accommodations, as these needs constantly evolve during the recovery process. Furthermore, it is essential that accommodations genuinely support the employee. A lack of appropriate workplace adjustments can hinder an individual’s ability to maintain employment, potentially negatively affecting their overall quality of life [8]. As shown in our participants’ stories, managers who understood cancer survivors’ challenges with RTW supported them in RTW more easily. Managers who are inflexible and unwilling to accept their employees’ suggestions may worsen the challenges employees face in RTW. To improve support, managers should have training to engage in RTW conversations, offer flexible scheduling, and adjust workloads to align with survivors’ capacities. Furthermore, workplaces and organizations could partner with employee assistance services to provide health and well-being solutions for their employees. Employers and managers play a crucial role in RTW experiences and must ensure they meet the needs of cancer survivors before, during, and after RTW.
