# Correction: Redefining ‘normal’: A Canadian case study of cancer survivors’ experiences remaining and/or returning to work during the COVID-19 pandemic

**DOI:** 10.1371/journal.pone.0347164

**Published:** 2026-04-10

**Authors:** The PLOS One Staff

## Notice of Republication

This article was republished on 27^th^ March 2026, to correct an error in XML. The subheadings “Patient self-advocacy” and “The need for flexible workplace accommodations” were formatted at the incorrect hierarchy level and should appear as subheading level 3. The publisher apologises for this error.
